# The effectiveness and safety of electroacupuncture for nonspecific chronic low back pain: A protocol for systematic review and/or meta-analysis

**DOI:** 10.1097/MD.0000000000024281

**Published:** 2021-01-29

**Authors:** Won-Suk Sung, Jeong Ryul Park, Kyungbok Park, Inae Youn, Hye Won Yeum, Sungyoon Kim, Jieun Choi, Yeeun Cho, Yejin Hong, Yeoncheol Park, Eun-Jung Kim, Dongwoo Nam

**Affiliations:** a Department of Acupuncture & Moxibustion, Dongguk University Bundang Oriental Hospital, Seongnam-si, Gyeonggi-do; b Department of Clinical Korean Medicine, Graduate School, Kyung Hee University; c Singil Kyunghee Korean Medical Clinic, Seoul; d Kyunghee Taerim Korean Medical Clinic, Incheon; e Department of Acupuncture & Moxibustion, Kyung Hee University Hospital at Gangdong; f Department of Acupuncture & Moxibustion, College of Korean Medicine, Kyung Hee University, Seoul, Republic of Korea.

**Keywords:** electroacupuncture, meta-analysis, non-specific chronic low back pain, systematic review

## Abstract

**Background:**

Low back pain (LBP) is a common symptom that affects almost 80% of the global population. LBP manifests as diverse pathologies and has different causes. The focus of this paper is nonspecific chronic low back pain (NSCLBP) wherein the pain lasts for more than 12 weeks, and for which there is no definite cause. Although there are various treatment options for NSCLBP, including medication and exercise, each option has its own limitations. Although electroacupuncture (EA) has been known to have useful analgesic effects on chronic LBP, there is no systematic review (SR) on EA in the literature. Therefore, this study aims to systematically review and validate the effectiveness and safety of EA for NSCLBP.

**Methods:**

We will search for randomized controlled trials on the use of EA for NSCLBP in multiple electronic databases, manual searches, and contacting authors. We will screen and select studies according to the predefined criteria and extract the data needed for this SR. The primary outcome will be the pain index (Visual Analog Scale and Numeric Rating Scale), and the secondary outcomes will be the functional status (Roland-Morris Disability Questionnaire), patient-centered outcomes, and adverse events. We will perform a meta-analysis using Review Manager software (Version 5.3; Copenhagen; The Nordic Cochrane Center, The Cochrane Collaboration, 2014) and assess the risk of bias using Cochrane Collaboration “risk of bias” tools and the quality of evidence using the Grades of Recommendation, Assessment, Development and Evaluation.

**Results:**

Our SR will investigate the effectiveness and safety of EA on NSCLBP.

**Conclusion:**

Our SR will support the published clinical evidence of the usage of EA for NSCLBP to assess the effectiveness and safety of EA.

**Trial registration number:**

INPLASY; INPLASY2020120039

## 1 Introduction

Low back pain (LBP) is a common disorder that affects nearly 80% of the global population and is associated with a number of causes such as fracture, radiculopathy, malignancy, and inflammatory disorder.^[1,2]^ However, when the cause of LBP cannot be determined because of the lack of detection of any pathoanatomic/radiologic abnormality, it is classified as nonspecific LBP.^[3]^ According to the duration, LBP can be classified into acute (<6 weeks), subacute (6–12 weeks), and chronic (>12 weeks) types.^[4]^ When the symptoms last for more than 12 weeks, and the cause of pain is unknown, the condition is referred to as nonspecific chronic low back pain (NSCLBP).

Although the exact prevalence of NSCLBP has not been determined, it can be inferred from existing reports. Some articles have reported that up to 60% of LBP patients did not recover fully by 12 weeks,^[5]^ and that nonspecific LBP accounts for 90% to 95% of LBP after excluding the possibility of spinal pathology and radicular syndrome.^[6]^ Thus, NSCLBP poses an economic burden by increasing the cost of treatment and having negative effects on work productivity, quality of life (QOL), as well as on social and psychological status of the affected individual.^[7]^

Although there are several therapies for NSCLBP, nonsteroidal anti-inflammatory drugs (NSAIDs) and exercise have been recommended in the 2018 Clinical Practice Guideline.^[8]^ However, NSAIDs are not recommended for long-term use because of their side effects on the renal, gastrointestinal, and cardiovascular systems.^[9,10]^ Combined with education, exercise is effective only in preventing LBP, with little evidence of its therapeutic effect.^[11]^ Other physical therapies, including transcutaneous electrical nerve stimulation and shock wave therapy, also have no significant effects.^[12,13]^

Because of the aforementioned dissatisfaction with conventional treatments, recent guidelines and systematic reviews (SRs) have recommended acupuncture as a cost-effective treatment for NSCLBP.^[14–16]^ Electroacupuncture (EA), a type of acupuncture, has been widely used for musculoskeletal disorders.^[17]^ Studies on the analgesic mechanism of EA have reported its association with endogenous opioid peptides. EA has been suggested as a therapeutic option for chronic pain to safely reduce the use of opioid medication and as an effective option for chronic LBP in combination with exercise.^[18,19]^ However, there are no SRs focusing on the use of EA for NSCLBP. Therefore, the purpose of this SR was to evaluate the effectiveness and safety of EA by examining published randomized controlled trials (RCTs) in the literature.

## 2 Methods

### 2.1 Study design

This SR will be conducted in accordance with the Preferred Reporting Items for Systemic reviews and Meta-Analyses Protocols (PRISMA-P) 2015 Statement.^[20]^

### 2.2 Ethics

No ethical statement is required, as there is no need for patient recruitment and personal data collection.

### 2.3 Study registration

The protocol was registered in INPLASY (Registration number: INPLASY2020120039).

### 2.4 Eligibility criteria

#### 2.4.1 Participants

Patients aged 18 years and more and diagnosed with NSCLBP will be included in this SR. LBP patients with pain lasting for more than 3 months without any pathological and anatomical abnormality will be included. We will exclude patients who have specific reasons for LBP including acute sprain, herniated disc, and fracture.

#### 2.4.2 Types of interventions

RCTs that investigated the effects of EA will be included in this study. The combination of conventional therapies will be accepted if the only difference between the groups is EA. Studies that used EA, but compared the treatment duration and different acupuncture points will be excluded.

#### 2.4.3 Type of studies

We will only include RCTs for the use of EA for NSCLBP. We will exclude non-RCTs including case reports, observational studies, cross-sectional studies, pilot studies, and SR. If the study did not describe the randomization method or used an incorrect method, it will be excluded from the SR.

#### 2.4.4 Outcome measures

Pain index (Visual Analog Scale and Numeric Rating Scale) will be the primary outcome measure. Secondary outcome measures will include functional status (Roland-Morris Disability Questionnaire), patient-centered outcomes, and adverse events.

#### 2.4.5 Language

There will be no limits on the language.

### 2.5 Information sources and search strategy

The following electronic databases will be used from their inception to May 2021: MEDLINE, EMBASE, Cochrane library, China National Knowledge Infrastructure (CNKI), CiNii, J-STAGE (Japanese database), KoreaMed, Korean Medical Database, Korean Studies Information Service System (KISS), National Digital Science Library (NDSL), Korea Institute of Science and Technology Information (KISTI), and Oriental Medicine Advanced Searching Integrated System (OASIS). Researchers will perform a search using terms with a combination of LBP-related terms (such as LBP, sciatica, backache, lumbago) and treatments (acupuncture, electroacupuncture) in each database's own language. If necessary, a manual search will also be carried out to include textbooks and references on EA, and the corresponding authors will be contacted (Table 1).

**Table 1:** Search strategy for the EMBASE.

No.	Search terms
#1	’Low Back Pain’/exp
#2	'Sciatica’/exp
#3	’Radiculopathy’/exp
#4	((lumbar OR lumbosacral OR ’lumbo sacral’ OR back) NEAR/5 (pain^∗^ OR ache^∗^ OR aching)):ab,ti
#5	(backache^∗^ or lumbago or sciatica):ab,ti
#6	(radiculopathy or radiculitis or radicular pain^∗^):ab,ti
#7	(nerve root^∗^ NEAR/5 (pain^∗^ or avulsion or compress^∗^ or disorder^∗^ or pinch^∗^ or inflam^∗^ or imping^∗^ or irritat^∗^ or entrap^∗^ or trap^∗^)):ab,ti
#8	#6 OR #7
#9	(back^∗^ or lumbosacral or lumbo-sacral or lumbar):ab,ti
#10	#8 and #9
#11	#1 OR #2 OR #3 OR #4 OR #5 OR #10
#12	’Acupuncture’/exp
#13	’Acupuncture Therapy’/exp
#14	’Acupuncture Point’/exp
#15	(acupuncture or electro-acupuncture or electroacupuncture or acupressure or needling or needle):ab,ti
#16	(acupoint^∗^ or meridian^∗^):ab,ti
#17	#12 OR #13 OR #14 OR #15 OR #16

### 2.6 Study selection

**Figure 1:**
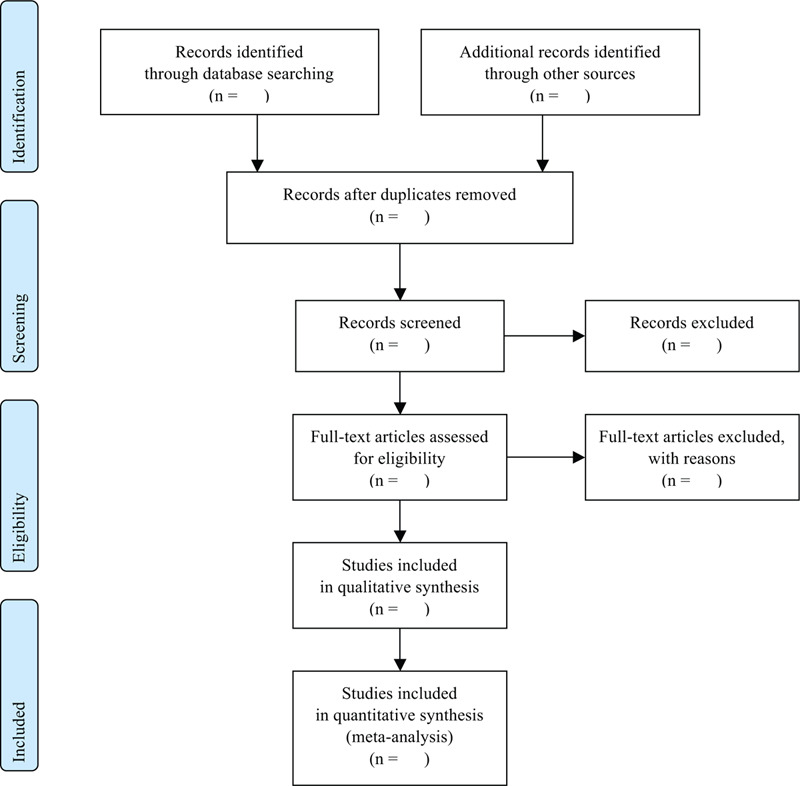
PRISMA flow diagram.

Two researchers will identify the titles, abstracts, and full-text (if possible) and screen the studies. After excluding the duplicates and irrelevant articles, the 2 researchers will review the studies by reading the full-texts and confirm the eligibility. Disagreement between the 2 researchers will be resolved by the discussion. If necessary, a third reviewer will mediate the discussion (Fig. 1).

### 2.7 Data management

Researchers will manage the studies using Endnote X9.

### 2.8 Data extraction

Two reviewers will extract information about the first author, publication year, the characteristics of the patients and control, the intervention characteristics (process, acupuncture points, and period), outcome measures, results, and study quality. Any disagreement between the 2 reviewers will be resolved by discussion or additional mediation as indicated above. If relevant data are not usable, we will contact the original author to obtain the missing data by e-mail. If the author or data are not available, we will mention this in the SR for that particular paper.

### 2.9 Data synthesis and analysis

The changes between the baseline and the completion of the intervention in the included studies will be used for the meta-analysis by using Review Manager software (Version 5.3; Copenhagen; The Nordic Cochrane Center, The Cochrane Collaboration, 2014). If the outcome measures are the same, the mean difference and 95% confidence intervals (CIs) will be calculated. For the different outcome measures, the standardized mean difference and 95% CI will be calculated to estimate the effect.

The statistical heterogeneity between the studies will be determined by Chi-squared and *I*-squared tests. The interpretation of the heterogeneity will be as follows:

*I*-squared: 0% to 40% unimportant heterogeneity; 30% to 60% moderate heterogeneity; 50% to 90% substantial heterogeneity; and 75% to 100% considerable heterogeneity. If possible, subgroup analysis will be conducted based on the control interventions. If quantitative synthesis is not possible, a narrative synthesis will be performed based on the available data.

Publication bias will be assessed by using the funnel plot if more than 10 identified studies are included in the meta-analysis. For determining the quality of evidence, the Grading of Recommendations Assessment, Development and Evaluation (GRADE) method will be utilized.^[21]^

### 2.10 Risk of bias assessment

Two reviewers will conduct the assessment of risk of bias. They will use the Cochrane Collaboration “risk of bias,” which consists of 6 domains (sequence generation, allocation concealment, blinding of participants, blinding of outcome assessors, incomplete outcome data, and selective outcome reporting).^[22]^ Two reviewers will independently assess the risk of bias, and any disagreements will be resolved through discussion. If consensus is not reached, a third party will participate in mediation.

## 3 Discussion

NSCLBP is a condition that affects the QOL by causing persistent pain. Although conventional treatments are helpful, they have their limitations; hence, acupuncture has been suggested as an alternative option for chronic LBP. EA, a type of acupuncture, could be helpful for NSCLBP; however, no SR has not been published on the use of EA for NSCLBP. Our study will provide the clinical evidence for the effectiveness and safety of EA on NSCLBP, which should be useful as a resource for health policy makers, practitioners, patients, and researchers.

## Author contributions

**Conceptualization:** Won-Suk Sung, Eun-Jung Kim.

**Funding acquisition:** Dongwoo Nam.

**Investigation:** Kyungbok Park, Inae Youn, Hye Won Yeum, Sungyoon Kim.

**Methodology:** Jeong Ryul Park, Yejin Hong.

**Project administration:** Yeoncheol Park, Eun-Jung Kim, Dongwoo Nam.

**Supervision:** Dongwoo Nam.

**Writing – original draft:** Jeong Ryul Park, Won-Suk Sung.

**Writing – review & editing:** Jieun Choi, Yeeun Cho, Yejin Hong, Won-Suk Sung, Dongwoo Nam.

## Abbreviations

Abbreviations: CI = confidence intervals, EA = electroacupuncture, LBP = low back pain, NSAID = nonsteroidal anti-inflammatory drugs, NSCLBP = nonspecific chronic low back pain, QOL = quality of life, RCT = randomized clinical trial, SR = systematic review.
